# Efficient One‐Pot Synthesis of 1,2,4,5‐Tetrasubstituted Imidazoles Using GO@SiO_2_@[(CH_2_)_3_Im][Cl] as a Novel Nanocomposite Under MW Irradiation

**DOI:** 10.1002/open.70281

**Published:** 2026-08-24

**Authors:** Mohamed Abu Shuheil, Afaq Mahdi Ali, Mekkanti Manasa Rekha, Subhashree Ray, Talal Aziz Qassem, T. Krithiga, Renu Sharma, Prakhar Tomar, Reza Mohammadi

**Affiliations:** ^1^ Faculty of Allied Medical Sciences Hourani Center for Applied Scientific Research Al‐Ahliyya Amman University Amman Jordan; ^2^ College of Pharmacy Department of Pharmaceutical Sciences AL‐Turath University Baghdad Iraq; ^3^ Department of Chemistry and Biochemistry School of Sciences JAIN (Deemed to be University) Bangalore Karnataka India; ^4^ Department of Biochemistry IMS and SUM Hospital Siksha ‘O’ Anusandhan Bhubaneswar Odisha India; ^5^ Department of Medical Laboratory Technics College of Health and Medical Technology Alnoor University Mosul Iraq; ^6^ Department of Chemistry Sathyabama Institute of Science and Technology Chennai Tamil Nadu India; ^7^ Department of Chemistry University Institute of Sciences Chandigarh University Mohali Punjab India; ^8^ Centre for Research Impact & Outcome Chitkara University Institute of Engineering and Technology Chitkara University Rajpura Punjab India; ^9^ Young Researchers and Elite Club Tehran Branch Islamic Azad University Tehran Iran

**Keywords:** green chemistry, microwave, multicomponent reactions, nanocomposite catalyst, solvent‐free, synthesis, tetrasubstituted imidazoles

## Abstract

This study presents the development of a novel nanocomposite catalyst, GO@SiO_2_@[(CH_2_)_3_Im][Cl], for the efficient one‐pot synthesis of 1,2,4,5‐tetrasubstituted imidazoles under environmentally benign conditions. The catalyst design incorporates graphene oxide, silica, and an imidazolium‐functionalized layer, creating a unique catalytic environment for the multicomponent reaction. Thorough characterization of the catalyst was performed using various analytical techniques, including FT‐IR, XRD, SEM, TEM, and BET surface area analysis, confirming its successful synthesis and structural integrity. Under optimized solvent‐free conditions and microwave irradiation, the reaction proceeds rapidly at room temperature, achieving excellent yields (90%–98%). Notably, the catalyst could be reused for up to seven consecutive cycles with minimal loss of activity and exhibits remarkable efficiency in promoting the four‐component condensation of ammonium acetate, a substituted aniline, a substituted benzaldehyde, and benzil to form highly functionalized imidazoles in 18 examples. This sustainable approach represents a significant advancement over traditional methods, which often require harsh conditions, extended reaction times, and generate substantial waste. The use of microwave irradiation, coupled with a solvent‐free protocol and room‐temperature conditions, aligns perfectly with green chemistry principles. This work provides a practical and sustainable route to important heterocyclic scaffolds. It highlights the potential of rationally designed nanocomposite catalysts in developing more environmentally friendly synthetic processes for complex organic transformations.

## Graphical abstract

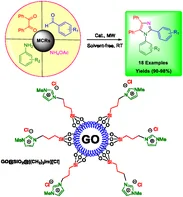

## 1 Introduction

The imidazole ring is a foundational motif in medicinal chemistry, present in natural products, pharmaceuticals, and a wide array of bioactive compounds. This five‐membered aromatic heterocycle, with two nitrogens and three carbons, imparts distinctive physicochemical properties—amphoterism, strong hydrogen‐bonding capacity, and a tendency to coordinate metal ions—that collectively enhance its versatility in drug discovery [1, 2, 3, 4]. L‐Histidine underscores the relevance of the imidazole nucleus by serving as a precursor to histamine and participating in numerous physiological processes; the imidazole core also features prominently in purine bases and many natural products. Within this family, 1,2,4,5‐tetrasubstituted imidazoles have emerged as particularly impactful. Fully substituted at all ring positions, these derivatives exhibit reduced tautomeric mobility, translating into greater stability, altered electronic properties, improved pharmacokinetics, and heightened selectivity in biological interactions [5, 6, 7, 8]. Literature surveys consistently reveal that this scaffold supports a broad spectrum of pharmacological and biological activities, including antibacterial [9], antihypertensive [10], anti‐tubercular [11], antiviral [12], anticoagulants [13], anti‐inflammatory [14], anti‐cancer [15], analgesic [16], inhibitor [17], antifungal [18, 19], and antibacterial activities [20]. Beyond pharmaceutical applications, 1,2,4,5‐tetrasubstituted imidazoles serve as robust ligands in coordination chemistry [21, 22], as potential fungicides or insecticides in agrochemistry [23, 24, 25], and in organic electronics and dyes due to their electron‐rich aromatic framework [26, 27, 28].

The substrate scope of imidazole synthesis via multicomponent reactions, particularly the Debus–Radziszewski condensation, demonstrates considerable versatility across a range of building blocks when appropriate catalysts and conditions are employed. Aromatic aldehydes are generally the most reliable partners and afford the corresponding 2,4,5‐trisubstituted imidazoles in good to excellent yields, with both electron‐donating and electron‐withdrawing substituents on the ring being well tolerated; electron‐withdrawing groups often accelerate the reaction by increasing electrophilicity, while halogenated substrates provide convenient handles for further functionalization [29, 30, 31]. Ortho‐substitution introduces steric hindrance that can slow condensation and cyclodehydration, sometimes necessitating extended reaction times or modified conditions. In contrast, heteroaromatic aldehydes are competent participants but may require careful catalyst selection to avoid coordination or deactivation. Aliphatic and α,β‐unsaturated aldehydes are also accessible. However, they tend to be less reactive than their aromatic counterparts, demand more careful handling due to volatility and a propensity for side reactions such as enolization or polymerization, and often benefit from judicious control of temperature and acidity. For the 1,2‐dicarbonyl component, symmetrical diketones such as benzil serve as benchmarks for constructing 4,5‐diaryl frameworks, yet glyoxal, biacetyl, and unsymmetrical or mixed analogs expand the available substitution patterns at the 4‐ and 5‐positions. However, the latter may exhibit reduced reactivity and profit from microwave assistance or stronger Brønsted or Lewis acid catalysis [32, 33, 34]. Nitrogen sources offer additional flexibility: ammonium acetate efficiently delivers *N*‐unsubstituted imidazoles, while primary amines furnish *N*‐substituted derivatives, with less hindered aliphatic amines reacting more readily than anilines and sterically encumbered amines typically requiring higher temperatures or longer times; this variation is especially valuable for preparing ionic liquids and compound libraries. Across these components, a wide array of functional groups—including nitriles, trifluoromethyl, cyano, and ester moieties—are commonly tolerated. However, free carboxylic acids, highly basic sites, or strongly coordinating heteroatoms may attenuate catalyst activity and occasionally call for protecting groups or adjusted protocols. Recent advances employing microwave irradiation, nanocatalysts, heterogeneous acids, or solvent‐free conditions have further enhanced functional group tolerance and reaction efficiency, enabling the rapid assembly of libraries of highly functionalized tri‐ and tetrasubstituted imidazoles suitable for pharmaceutical and materials applications and underscoring the enduring value of this multicomponent reaction as a robust, atom‐economical platform [35, 36, 37, 38].

A central objective in modern methodology is the design of greener, more sustainable protocols. Classical routes often require elevated temperatures, long reaction times, and volatile organic solvents, contributing to energy consumption and waste. The twelve principles of green chemistry, as articulated by Anastas and Warner, provide a framework for minimizing the use of hazardous substances, maximizing atom economy, using safer solvents, improving energy efficiency, and pursuing inherently safer chemistries. In heterocyclic synthesis, these considerations are particularly pressing given the traditional reliance on harsh conditions and solvent‐intensive workflows [39, 40, 41, 42].

Microwave irradiation has transformed the efficiency of multicomponent reaction (MCR) synthesis by enabling rapid energy transfer. Unlike traditional thermal methods that rely on slow conductive heating, microwaves deliver energy directly to polar molecules and ionic intermediates through dielectric heating. This results in almost instant, uniform volumetric heating, eliminating thermal gradients and significantly speeding up molecular kinetics. As a result, reactions that once took hours under reflux can now be completed in minutes, highlighting the practical advantages of microwave‐assisted methods in organic synthesis [43, 44, 45].

Beyond sheer speed, the synergy between microwave irradiation and green chemistry principles elevates its value for sustainable synthesis, particularly for MCRs. The technique is highly compatible with solvent‐free or near‐solventless conditions, addressing a major source of chemical waste and energy consumption in conventional organic synthesis. Eliminating volatile organic solvents not only simplifies work‐up procedures but also reduces environmental and safety hazards. Moreover, when combined with heterogeneous catalytic systems, the localized superheating effect at solid catalyst surfaces can further intensify reaction rates and selectivity, facilitating easier catalyst recovery and product isolation. Thus, by integrating rapid, directed energy input with inherently cleaner reaction designs, microwave‐assisted synthesis provides a powerful and holistic platform. It advances MCR methodologies by making them not only faster and more efficient but also more aligned with the imperative of sustainable chemical development [46, 47].

The use of heterogeneous catalysts further intensifies the green credentials of microwave‐assisted, solvent‐free protocols. Reusable solid catalysts can be separated by straightforward filtration or centrifugation, enabling multiple reuse cycles with minimal loss of activity [48, 49, 50, 51]. When these catalysts are based on earth‐abundant, non‐toxic materials and prepared via energy‐efficient methods, their sustainability merits are amplified. Nanocomposite catalysts, which integrate multiple functional components at the nanoscale, have shown particular promise. By combining synergistic components, these materials can achieve superior activity, selectivity, stability, and recoverability compared with traditional catalysts [52, 53, 54, 55, 56].

Graphene oxide is a versatile platform for constructing multifunctional nanocomposite catalysts in this context. Its two‐dimensional, atomically thin structure, rich in oxygen‐containing functional groups, provides an expansive surface area and abundant anchoring sites for immobilizing catalytic species [57, 58, 59]. Epoxy, hydroxyl, carbonyl, and carboxyl groups on graphene oxide enable covalent or non‐covalent attachment of diverse catalytic moieties and facilitate interactions such as π–π stacking with aromatic substrates, thereby helping pre‐concentrate substrates at the catalytic interface. Moreover, graphene oxide can facilitate localized microwave heating at active sites, thereby enhancing reaction rates while keeping the bulk medium at or near ambient temperature [60, 61, 62, 63].

Incorporating silica into nanocomposites adds mechanical robustness and provides a versatile platform for further functionalization through well‐established silane chemistry. Silica coatings protect the graphene oxide surface, prevent sheet aggregation, and offer abundant silanol groups for attaching catalytic sites or ionic liquid functionalities with precise control over coverage and distribution [64, 65, 66, 67]. Ionic liquids, particularly imidazolium‐based variants, contribute negligible vapor pressure, high thermal stability, and tunable polarity, enabling the immobilization of ionic liquids on solid supports to combine the advantages of homogeneous‐like catalytic environments with heterogeneous ease of use. The immobilized ionic liquid can function as a dual‐activation medium in multicomponent condensations, further stabilizing intermediates through hydrogen bonding or Lewis acid interactions and facilitating nucleophilic attack by stabilizing developing charges [68, 69, 70, 71, 72].

The strategic integration of graphene oxide, silica, and ionic liquid moieties within a single nanocomposite creates a catalytic system that embodies true synergy. Each component contributes distinct benefits: graphene oxide provides a high‐surface‐area, aromatic‐friendly interface that can attract substrates; silica offers structural stability and a versatile platform for functionalization; ionic liquids impart catalytic activity and substrate activation. Their intimate three‐way integration creates a microenvironment that supports cooperative activation pathways that are unlikely to be realized with simpler catalysts. The ionic liquid layer confined within the nanocomposite may exhibit properties different from those of bulk ionic liquids, potentially enhancing catalytic performance [73].

This study aims to develop and evaluate a green, operationally simple, and highly efficient protocol for the one‐pot synthesis of 1,2,4,5‐tetrasubstituted imidazoles using GO@SiO_2_@[(CH_2_)_3_Im][Cl] as a novel nanocomposite catalyst under microwave irradiation. Specifically, the research focuses on the four‐component condensation of ammonium acetate, substituted anilines, substituted benzaldehydes, and benzil under solvent‐free conditions at room temperature (RT), targeting very short reaction times and high isolated yields (90%–98%) across a representative library of 18 examples. Within this scope, the work seeks to demonstrate not only the catalytic efficiency and generality of the GO@SiO_2_@[(CH_2_)_3_Im][Cl] system toward structurally diverse substrates, but also its compliance with the principles of green chemistry, including energy efficiency, solvent avoidance, and waste minimization. In addition, the research examines the robustness and recyclability of the nanocomposite, the influence of electronic and steric effects of aromatic substituents on the reaction outcome, and the synthetic practicality of the protocol as a convenient route to pharmacologically relevant tetrasubstituted imidazole scaffolds.

## 2 Result and Discussion

**SCHEME 1:**
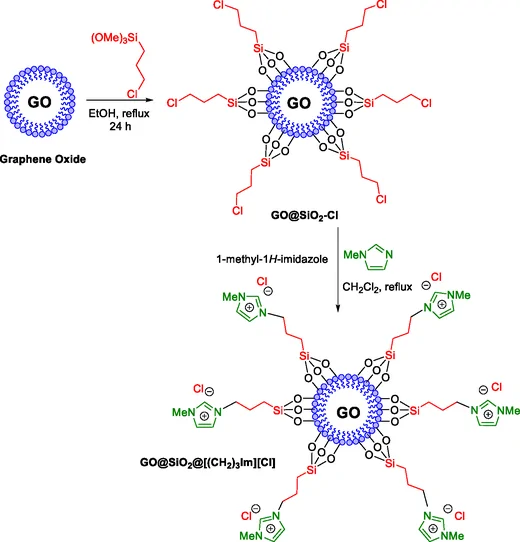
Structural preparation of GO@SiO_2_@[(CH_2_)_3_Im][Cl] nanocomposite.

The GO@SiO_2_@[(CH_2_)_3_Im][Cl] nanocomposite was synthesized following the pathway outlined in Scheme 1. Graphene oxide (GO) was first dispersed in ethanol by sonication and then treated with 3‐chloropropyltrimethoxysilane; the mixture was refluxed with stirring for 24 h to graft chloropropyl‐silane moieties and generate the chloropropyl‐functionalized silica‐coated material (GO@SiO_2_‐Cl). The solid was recovered by filtration (or centrifugation), washed thoroughly with ethanol to remove unreacted silane and by‐products, and dried. For introduction of the ionic liquid functionality, GO@SiO_2_‐Cl was suspended in dichloromethane and reacted with 1‐methyl‐1*H*‐imidazole under reflux to effect nucleophilic substitution (quaternization) of the surface‐bound chloropropyl groups, yielding the imidazolium chloride‐functionalized nanocomposite (GO@SiO_2_@[(CH_2_)_3_Im][Cl]). The product was isolated, washed with dichloromethane and ethanol to remove excess or physiosorbed reagents, and dried under vacuum prior to characterization.

To establish a comprehensive understanding of its structural and compositional properties, the resulting nanocatalyst was subjected to a detailed multi‐technique characterization. The morphology and spatial distribution of the material were examined using FT‐IR, SEM, TEM, XRD, TGA, and EDX to confirm the homogeneous dispersion of constituent elements. Together, this suite of analytical techniques provides a rigorous foundation for the material's confirmed architecture and subsequent catalytic applications.

### 2.1 Characterization of GO@SiO_2_@[(CH_2_)_3_Im][Cl] Nanocatalyst

**FIGURE 1:**
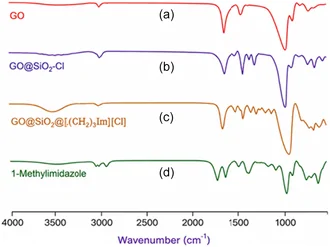
FT‐IR spectrums of (a) GO, (b) GO@SiO_2_‐Cl, (c) GO@SiO_2_@[(CH_2_)_3_Im][Cl], and (d) 1‐Methylimidazole.

The chemical composition and surface functionalization of the synthesized materials were characterized using Fourier‐transform infrared (FT‐IR) spectroscopy, as illustrated in Figure 1. The spectrum for graphene oxide (GO), labeled as trace (a), exhibits characteristic absorption bands including a broad peak around 3400 cm^−1^ corresponding to O–H stretching vibrations and a prominent peak near 1720 cm^−1^ attributed to the C=O stretching of carboxylic groups. The skeletal vibrations of the unoxidized graphitic domains are visible at approximately 1620 cm^−1^, while the intense band centered at 1050 cm^−1^ is indicative of C–O alkoxy or epoxy stretching, confirming the high degree of oxygen‐containing functional groups present on the GO sheets.

Upon the modification of graphene oxide with a silica shell to form GO@SiO_2_‐Cl, shown in trace (b), significant spectroscopic changes occur that confirm successful surface coating. The most notable feature is the intensification and broadening of the band in the 1000–1100 cm^−1^ region, which is characteristic of the asymmetric stretching vibrations of Si–O–Si linkages. Additionally, a distinct peak appears near 800 cm^−1^, corresponding to the Si–O bending vibration. The preservation of the carbonyl peak from the GO core suggests that the silica shell encapsulates the graphene structure without completely masking its primary oxygenated features.

The subsequent functionalization to produce GO@SiO_2_@[(CH_2_)_3_Im][Cl], represented in trace (c), introduces new vibrational modes that signify the grafting of the ionic liquid moiety. In this spectrum, the reappearance of aliphatic C–H stretching bands just below 3000 cm^−1^ indicates the presence of the propyl linkage. More importantly, several new absorption bands appear in the 1400–1600 cm^−1^ range, which correlate with the C=N and C=C skeletal vibrations of the imidazole ring. A comparison with the reference spectrum of 1‐methylimidazole in trace (d) confirms these assignments, as many of the fingerprint region peaks found in the pure imidazole—specifically those between 1100 and 1500 cm^−1^—are mirrored in the spectrum of the final composite material.

The evolution of these spectra provides clear evidence of the stepwise assembly of the nanocomposite. The transition from the oxygen‐rich GO to the silica‐coated GO@SiO_2_‐Cl is marked by the dominance of silicon‐oxygen vibrations, while the final immobilization of the imidazolium‐based ionic liquid is confirmed by the emergence of characteristic heterocyclic and alkyl signals. The successful integration of these components is evidenced by the overlap of the silica backbone's broad Si–O–Si stretch with the distinct organic signatures of the imidazole ring, confirming that the functionalized ionic liquid has been covalently tethered to the silica‐coated graphene framework.

A comparative analysis of the four FT‐IR spectra reveals a clear and logical progression of chemical transformations, each step building upon the previous one. The initial spectrum of graphene oxide (trace a) serves as the foundational reference, characterized by a high density of oxygen functionalities. This is evident from the strong O–H, C=O, and C–O stretches, which collectively define the highly oxidized and hydrophilic nature of the GO surface. This spectrum is the baseline against which all subsequent modifications are measured.

The transition to the silica‐coated intermediate (trace b) represents a fundamental shift in the material's surface chemistry. The most dramatic change is the emergence of the intense, broad Si–O–Si stretching band, which effectively dominates the mid‐infrared region. This new feature, along with the Si–O bending peak, indicates that the surface has been successfully passivated with a silica layer. Crucially, the persistence of the GO's carbonyl peak suggests that the coating is not a thick, opaque shell but rather a conformal layer that allows the underlying GO structure to still be detected. This step is therefore characterized by the addition of an inorganic silicate framework onto the organic GO template.

The final functionalization step (trace c) introduces a distinctly organic character back into the material, but of a different nature than the original GO. The appearance of aliphatic C–H stretches and, more importantly, the imidazole ring vibrations in the 1400–1600 cm^−1^ region, signals the successful grafting of the ionic liquid. The strength of this analysis is greatly enhanced by the inclusion of the pure 1‐methylimidazole reference spectrum (trace d). The direct spectral overlap between trace (c) and trace (d) in the fingerprint region provides unambiguous evidence that the imidazolium cation is indeed present in the final composite. This comparison eliminates ambiguity, confirming that the new peaks are not artifacts or degradation products but are directly attributable to the targeted ionic liquid structure.

In summary, the comparative analysis of these spectra tells a coherent story of a layer‐by‐layer assembly. The initial oxygen‐rich GO is first coated with an inorganic silica shell, which is then used as a platform for the covalent attachment of an organic ionic liquid. The final spectrum of GO@SiO_2_@[(CH_2_)_3_Im][Cl] is not merely a sum of its parts but a unique composite signature, where the broad Si–O–Si backbone of the silica shell coexists with the sharp, characteristic signals of the imidazole ring and the alkyl linker. This confirms the successful synthesis of a hybrid organic‐inorganic nanocomposite with a well‐defined chemical structure.

**FIGURE 2:**
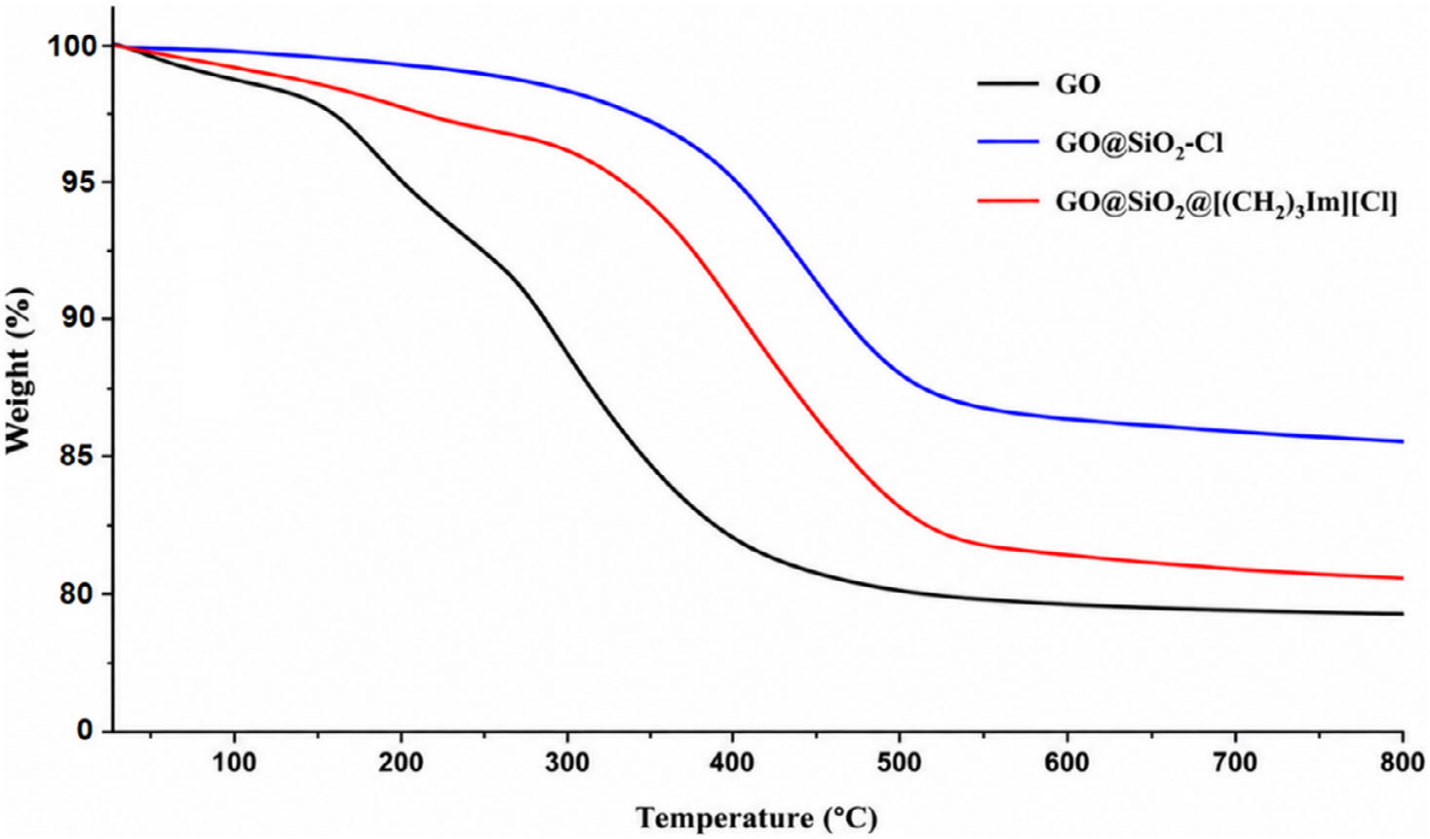
TGA analysis of GO@SiO_2_@[(CH_2_)_3_Im][Cl] catalyst.

The thermal stability and decomposition profiles of the synthesized materials were evaluated using thermogravimetric analysis (TGA) under an inert atmosphere, with the results depicted in Figure 2. The TGA curves illustrate the weight loss percentages as a function of temperature, ranging from ambient conditions to 800 °C, for graphene oxide (GO), silica‐functionalized graphene oxide (GO@SiO_2_‐Cl), and the final imidazolium‐based ionic liquid catalyst (GO@SiO_2_@[(CH_2_)_3_Im][Cl]).

The thermogram for pristine GO, represented by the black curve, exhibits a multi‐stage degradation process characteristic of carbonaceous materials with high oxygen content. An initial weight loss observed below 150 °C is attributed to the evaporation of physically adsorbed water molecules and moisture trapped within the graphene layers. A more pronounced and rapid weight loss occurs between 200 and 350 °C, during which approximately 15% of the mass is lost. This significant degradation is primarily due to the decomposition of labile oxygen‐containing functional groups, such as hydroxyl, epoxy, and carboxylic acid groups, resulting in the evolution of CO and CO_2_ gases. Beyond 500 °C, the weight continues to decrease at a slower rate due to the further carbonization of the graphene framework, eventually stabilizing with a total weight loss of roughly 21% at 800 °C.

In contrast, the GO@SiO_2_‐Cl intermediate, shown by the blue curve, demonstrates significantly enhanced thermal stability. The initial decomposition stage seen in pure GO is largely mitigated, indicating that the silica coating acts as a protective barrier and that many of the oxygen functional groups on the GO surface were consumed or shielded during the silanization process. The major weight loss for this material begins at a much higher temperature, approximately 350 °C, and levels off around 550 °C. The residual mass of approximately 85% at 800 °C—compared to the 79% residue for pure GO—confirms the successful incorporation of the thermally stable inorganic silica layer.

The final catalyst, GO@SiO_2_@[(CH_2_)_3_Im][Cl], represented by the red curve, shows a distinct decomposition pattern that lies between the precursor materials. While it maintains better stability than pure GO at lower temperatures, it exhibits an additional weight loss stage starting around 300 °C. This specific mass loss, which accounts for an approximate 5% difference compared to the GO@SiO_2_‐Cl precursor, is attributed to the thermal degradation of the organic imidazolium ionic liquid moieties grafted onto the silica surface. The decomposition of these organic chains continues until approximately 550 °C, after which the curve plateaus, leaving a final residue of about 80.5%.

A comparative analysis of these thermograms provides strong evidence for the successful sequential functionalization of the graphene oxide. The shift in decomposition temperatures and the variations in final residual weights confirm the transition from pure GO to a silica‐coated intermediate, and finally to the organic–inorganic hybrid catalyst. The data suggests that while the introduction of the ionic liquid reduces the overall thermal stability compared to the silica‐only intermediate due to the inherent volatility of organic components, the final catalyst remains thermally robust up to 300 °C, making it highly suitable for a wide range of catalytic applications in organic synthesis.

**FIGURE 3:**
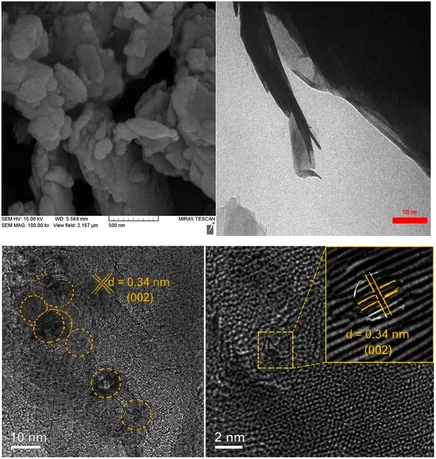
SEM, TEM images, and HR‐TEM mapping analysis of GO@SiO_2_@[(CH_2_)_3_Im][Cl] catalyst at different magnifications.

The morphological and structural characteristics of the GO@SiO_2_@[(CH_2_)_3_Im][Cl] catalyst were investigated using a combination of electron microscopy techniques (Figure 3). Scanning electron microscopy (SEM), performed at a high magnification of 100.00 k× with an accelerating voltage of 15.00 kV, revealed a complex, irregular surface architecture consisting of aggregated, flake‐like structures. These features are characteristic of functionalized graphene oxide, where the initial planar sheets have undergone significant topographical modification due to the multi‐step grafting of the silica layer and the subsequent immobilization of the imidazolium‐based ionic liquid.

Further insights into the internal structure and layering of the material were provided by transmission electron microscopy (TEM). The micrographs illustrated the exfoliated nature of the composite, showing distinct, semi‐transparent layers that overlap to form a porous matrix. The presence of these thin, sheet‐like morphologies confirmed that the graphene oxide framework remains largely intact as a foundational support, providing a high surface area for the distribution of the catalytic sites.

To evaluate the material at the atomic level, high‐resolution transmission electron microscopy (HR‐TEM) was employed (Figure 3). At a 10‐nm scale, the material exhibited a highly textured surface with visible lattice fringes, suggesting a degree of crystallinity within the carbonaceous framework. The presence of nanostructured domains, highlighted by dashed circles, indicated the successful incorporation of functional groups or clusters across the graphene oxide substrate. A more detailed lattice analysis at the 2‐nm scale identified a prominent interplanar spacing (d‐spacing) of 0.34 nm. This measurement corresponds precisely to the (002) crystal plane of graphitic carbon, signifying the preservation of the hexagonal carbon lattice despite the extensive chemical functionalization.

When comparing these observations, a clear correlation emerges between the micro‐scale surface roughness seen in the SEM and the nano‐scale layering observed in the TEM and HR‐TEM. The macroscopic aggregation of flakes observed in SEM results from the stacking and intertwining of the individual sheets captured in the TEM images. Crucially, the HR‐TEM data provides the definitive evidence required to bridge these observations, confirming that while the overall morphology is heavily modified by the silica and ionic liquid coating, the underlying graphitic order—characterized by the 0.34 nm d‐spacing—remains a robust structural constant in the GO@SiO_2_@[(CH_2_)_3_Im][Cl] catalyst. This hierarchical structural integrity is vital for ensuring both mechanical stability and efficient catalytic performance in various chemical transformations.

The three imaging techniques employed in this study offer complementary perspectives that, when integrated, provide a comprehensive understanding of the catalyst's architecture. The SEM analysis serves as the macroscopic starting point, revealing a highly roughened and aggregated surface. This observation alone, however, cannot distinguish between simple particle clumping and a more complex, layered internal structure. The TEM analysis resolves this ambiguity by penetrating the surface to reveal the exfoliated, sheet‐like nature of the composite. It confirms that the “flakes” seen in SEM are not amorphous aggregates but rather overlapping, semi‐transparent graphene oxide sheets, which are responsible for the high surface area of the support.

The most critical link in this analytical chain is provided by HR‐TEM. While SEM and TEM describe the material's shape and layering, HR‐TEM probes its fundamental crystalline order. The detection of a 0.34 nm d‐spacing, corresponding to the (002) plane of graphite, is a definitive finding. It demonstrates that the rigorous chemical processes of silica grafting and ionic liquid immobilization, which dramatically alter the surface topography (as seen in SEM) and the sheet stacking (as seen in TEM), do not destroy the core graphitic backbone of the material. This preservation of the carbon lattice is essential, as it provides the mechanical robustness and electronic properties that underpin the catalyst's stability and activity. Therefore, the comparative analysis reveals a hierarchical structure where a heavily modified and functionalized surface coats a structurally pristine and ordered graphitic core, a combination that is likely key to the material's catalytic function.

**FIGURE 4:**
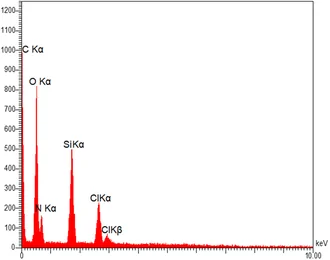
EDX analysis of GO@SiO_2_@[(CH_2_)_3_Im][Cl] catalyst.

The energy‐dispersive X‐ray (EDX) spectrum of the GO@SiO_2_@[(CH_2_)_3_Im][Cl] material displays the characteristic K‐lines of the constituent light elements. It confirms the presence of the functional groups introduced during catalyst preparation (Figure 4). Distinct peaks are observed at the following characteristic energies: C Kα (~0.28 keV), N Kα (~0.39 keV), O Kα (~0.52 keV), Si Kα (~1.74 keV), and two chlorine lines, Cl Kα (~2.62 keV) and the weaker Cl Kβ (~2.81 keV). The signal intensity is highest for the oxygen and carbon lines, followed by a clear silicon peak; nitrogen and chlorine are present at substantially lower intensity.

These assignments are consistent with the intended material composition. The pronounced C and O signals reflect the graphene oxide (GO) backbone and its oxygen‐containing functional groups. The Si Kα peak confirms the presence of the silica component (SiO_2_) used to coat or anchor the GO sheets. The detection of N Kα, although of low intensity, indicates incorporation of nitrogen‐containing organic functionality associated with the immobilized ionic liquid moiety (e.g., an imidazolium or related nitrogenous linker). The presence of both the Cl Kα and the weaker Cl Kβ lines provides direct evidence for chloride as the counterion of the immobilized ionic liquid. The relatively low intensities of N and Cl compared with C, O, and Si are expected for a surface‐modified material in which the ionic‐liquid‐derived species are present as a thin surface layer or grafted groups rather than as a bulk component.

No additional elemental peaks (e.g., transition metals or other heteroatoms) are apparent in the spectral window shown (0–10 keV), supporting the absence of metal contamination and confirming that the major constituents are C, O, Si, N, and Cl. It should be noted that EDX is semi‐quantitative and less sensitive to very light elements and to low surface coverages; peak intensities reflect both atomic concentration and X‐ray generation/detection efficiencies. Nevertheless, the observed elemental pattern and the presence of the diagnostic Cl Kα/Kβ pair together provide robust qualitative confirmation that the silica‐coated GO has been successfully functionalized with a nitrogen‐containing ionic‐liquid species bearing a chloride counterion.

**FIGURE 5:**
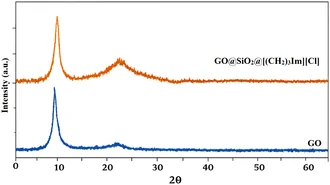
XRD analysis of GO NPs and GO@SiO_2_@[(CH_2_)_3_Im][Cl] catalyst.

Figure 5 presents the X‐ray diffraction (XRD) patterns collected for pristine graphene oxide nanoparticles (GO), and for the silica‐ and ionic‐liquid‐modified material denoted GO@SiO_2_@[(CH_2_)_3_Im][Cl]. The diffraction trace of GO is dominated by a sharp, well‐defined reflection at approximately 2*θ *= 10°, which is characteristic of the (001) spacing of oxidized graphitic layers. Using Cu Kα radiation (*λ *= 1.5406 Å), this peak corresponds to an interlayer d‐spacing on the order of 0.88 nm (*d *≈ 8.8 Å), consistent with oxygen functional groups and intercalated water molecules that expand the graphite interlayer distance in GO. Aside from this (001) reflection, the GO pattern shows only a weak, broad feature at higher angles, indicating limited short‐range order beyond the layer stacking.

After surface modification GO@SiO_2_@[(CH_2_)_3_Im][Cl], the XRD pattern retains a feature in the low‐angle region associated with the GO (001) reflection. However, its profile is altered: the reflection becomes less intense and significantly broadened, and an increased baseline partially obscures the low‐angle region. In addition, a broad diffuse scattering centered near 2*θ *≈ 20°–25° appears in the modified material. The broadness and lack of sharp peaks in this mid‐angle region are characteristic of amorphous silica and of a loss of long‐range order; the broad band at ~23° corresponds to an interplanar spacing of roughly 0.38–0.39 nm, a value comparable to short‐range graphitic (002) distances but here more plausibly assigned to the amorphous silica coating and to reduced stacking coherence of the carbon sheets.

Taken together, the comparative analysis of the two patterns indicates that the GO lattice integrity is preserved mainly after functionalization, in the sense that the (001) reflection remains observable, but that the ordering along the *c*‐axis is disrupted. The broadening and reduced intensity of the GO (001) peak in the GO@SiO_2_@[(CH_2_)_3_Im][Cl] sample are consistent with partial exfoliation or increased disorder of the layered structure and/or with disorder introduced by silica deposition and ionic liquid immobilization between or on the surfaces of the layers. The emergence of a broad feature centered at ~20°–25° and the absence of new sharp crystalline reflections demonstrate that the deposited silica is predominantly amorphous and that no crystalline impurity phases or crystalline salt aggregates are present at detectable levels. Thus, the XRD data support the formation of a core GO structure coated or decorated with amorphous SiO_2_ and an immobilized imidazolium chloride ionic liquid, producing diminished long‐range graphitic order while preserving the basic GO framework.

**FIGURE 6:**
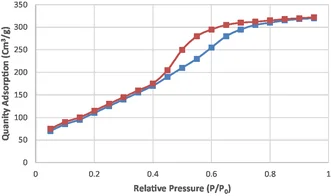
BET analysis of the GO@SiO_2_@[(CH_2_)_3_Im][Cl] nanocomposite.

Figure 6 presents the nitrogen adsorption–desorption isotherm of the GO@SiO_2_@[(CH_2_)_3_Im][Cl] nanocomposite, plotted as adsorbed volume (cm^3^/g) versus relative pressure (P/P0). At the lowest relative pressures, the adsorbed volume is relatively small, on the order of 70–90 cm^3^/g, and the adsorption and desorption branches are essentially coincident, consistent with limited micropore filling. Between P/P0 ≈ 0.1 and 0.4, the adsorbed amount increases approximately linearly from roughly 90–100 cm^3^/g to about 170–190 cm^3^/g, indicating multilayer adsorption on accessible surface area. A pronounced inflection occurs at intermediate relative pressures: from P/P0 ≈ 0.45–0.60, the adsorption branch rises steeply from roughly 180–200 cm^3^/g to approximately 250–300 cm^3^/g. The desorption branch follows a similar overall trend but lies consistently below the adsorption branch across this interval, with the most significant deviation (on the order of 30–50 cm^3^/g) around P/P0 ≈ 0.55. Above P/P0 ≈ 0.7, the two branches converge, and the adsorbed volume approaches a plateau near 310–330 cm^3^/g as P/P0 → 1.0.

The overall shape of the isotherm and the presence of a clear hysteresis loop between about P/P0 = 0.45 and 0.8 are characteristic of a Type IV isotherm, indicative of a mesoporous structure with capillary condensation occurring in the mesopore range. The hysteresis, with the desorption branch shifted to lower adsorbed volumes relative to adsorption, implies network effects or pore blocking and is commonly associated with slit‐like or plate‐like pore geometries that arise in layered materials; this is consistent with the expected architecture of a GO‐based composite decorated with silica and organic moieties. The steep uptake at intermediate relative pressures suggests a significant contribution from a dominant mesopore family that fills via capillary condensation. At the same time, the convergence of adsorption and desorption at high P/P0 indicates that larger meso‐ or macropores are also present and become filled near saturation.

From a functional standpoint, the high total uptake (≈310–330 cm^3^/g) and the mesoporous character inferred from the isotherm are advantageous for catalytic applications because they imply substantial pore volume and facile mass transport to interior active sites. The observed hysteresis signals a connected pore network but also suggests pore‐size heterogeneity or constrictions that could influence reactant/product diffusion during operation. The isotherm therefore supports the conclusion that grafting SiO_2_ and the imidazolium‐containing organic functionality onto the GO scaffold creates a porous composite that combines a large accessible surface area with mesopores suitable for liquid‐ and gas‐phase catalysis. At the same time, the specific shape and width of the hysteresis loop suggest pore geometries dominated by slit‐like openings between plate‐like components rather than solely cylindrical pores.

### 2.2 Zeta‐Potential Analysis

The evolution of the surface charge during nanocomposite synthesis was investigated by zeta‐potential measurements. The chlorinated silica‐coated graphene oxide intermediate, GO@SiO_2_–Cl, exhibited a predominantly negative surface potential, with the distribution centered at −22.6 mV and a maximum total count of approximately (5.0 × 10^5^). This negative charge can reasonably be associated with ionized oxygen‐containing groups on the graphene oxide framework and residual silanol groups on the silica coating. Although the material contains chloropropyl functionalities, the negative zeta potential should not be attributed solely to the electronegativity of chlorine because the measured value reflects the overall composition and ion distribution at the particle–liquid slipping plane.

Following functionalization with the imidazolium‐based ionic‐liquid moiety, the zeta‐potential distribution shifted markedly toward positive values. The final GO@SiO_2_@[(CH_2_)_3_Im][Cl] nanocomposite displayed a peak at +18.4 mV, corresponding to an overall change of 41.0 mV relative to the GO@SiO_2_–Cl intermediate. This charge reversal provides strong evidence that the surface chemistry was substantially altered during the final functionalization step. In particular, the positive potential is consistent with the presence of covalently immobilized imidazolium cations on the silica‐coated graphene oxide surface. These cationic groups compensate for and ultimately dominate the negative contributions of the underlying graphene oxide and silica layers, while chloride serves as the counterion. More precisely, the positive charge originates from the imidazolium functionality rather than from a conventional quaternary ammonium group.

Comparison of the two distributions therefore reveals an unambiguous transition from an anionic to a cationic particle interface. The magnitude of the zeta potential decreased slightly in absolute terms, from 22.6 mV for GO@SiO_2_–Cl to 18.4 mV for GO@SiO_2_@[(CH_2_)_3_Im][Cl], even though the sign was reversed. Consequently, the principal evidence for functionalization is the 41.0 mV displacement and reversal of charge rather than an increase in the absolute electrokinetic potential. The maximum count also increased from approximately (5.0 × 10^5^) to more than (5.4 × 10^5^). This difference may reflect variations in the number of detected scattering events, particle concentration, dispersion quality, or instrumental acquisition conditions; it should not, by itself, be interpreted as evidence of a greater surface charge or a higher degree of functionalization.

Both profiles appear relatively narrow and dominated by a single peak, suggesting that each dispersion contains a comparatively uniform population in terms of electrophoretic behavior. The absence of prominent secondary peaks is consistent with reasonably homogeneous surface modification, although zeta‐potential data alone cannot establish chemical homogeneity conclusively. Confirmation of covalent imidazolium grafting would ideally be supported by complementary characterization methods, such as X‐ray photoelectron spectroscopy, Fourier‐transform infrared spectroscopy, elemental analysis, or thermogravimetric analysis. Nevertheless, the observed reversal from −22.6 to +18.4 mV is strongly consistent with successful incorporation of the cationic imidazolium layer.

The positive surface potential of the final material may promote electrostatic attraction toward negatively charged substrates, biomolecules, catalyst supports, or ionic pollutants, potentially influencing adsorption and catalytic behavior. Its implications for colloidal stability, however, should be interpreted cautiously. Electrostatic stabilization generally increases with the absolute magnitude of the zeta potential, and values greater than approximately 30 mV in magnitude are often associated with strongly stabilized dispersions, although the relevant threshold depends on pH, ionic strength, solvent composition, and particle concentration. The measured value of +18.4 mV therefore indicates moderate electrostatic stabilization rather than exceptionally high colloidal stability. Moreover, because its absolute magnitude is lower than that of the precursor, the final material is not necessarily more stable solely on the basis of zeta potential. Dispersion stability should instead be verified through time‐dependent sedimentation, particle‐size, or aggregation measurements under the intended operating conditions.

Overall, the zeta‐potential results demonstrate a pronounced modification of the particle–solution interface during synthesis. GO@SiO_2_–Cl possesses a moderately negative surface charge, whereas immobilization of the imidazolium functionality produces a positively charged nanocomposite. This charge reversal supports the successful preparation of GO@SiO_2_@[(CH_2_)_3_Im][Cl] and indicates that the grafted ionic‐liquid layer is sufficiently abundant to govern the electrokinetic behavior of the composite. Finally, although the associated figure caption reportedly identifies the measurement as XPS analysis, the plotted potential distributions and count data are characteristic of electrophoretic light‐scattering or zeta‐potential measurements. The caption should therefore be corrected to avoid misidentification of the analytical technique.

**FIGURE 8:**
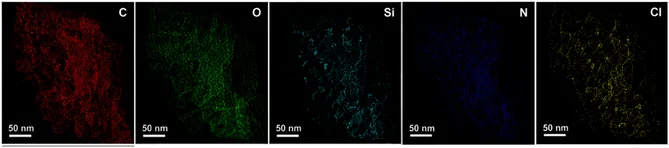
Elemental mapping analysis of the GO@SiO_2_@[(CH_2_)_3_Im][Cl] nanocomposite.

Figure 8 presents EDS elemental maps of the GO@SiO_2_@[(CH_2_)_3_Im][Cl] nanocomposite, showing broadly uniform distributions of C, O, Si, N, and Cl across the observed region at the 50 nm scale. The strong and extensive C and O signals are consistent with the graphene oxide framework and oxygen‐containing silica phase. In comparison, the Si distribution closely follows the carbon‐rich morphology, indicating that SiO_2_ is dispersed over the GO surface rather than segregated into distinct particles. The weaker N and Cl signals are nevertheless spatially distributed throughout the same region and overlap with the C and Si maps, supporting the presence of the imidazolium‐based ionic functionality on the silica‐coated GO.

Comparatively, the dominance of C and O reflects their greater abundance in the supporting framework, whereas the lower‐intensity N and Cl maps correspond to the surface‐bound ionic component. Their colocalization with Si suggests relatively homogeneous immobilization of [(CH_2_)_3_Im][Cl] on the SiO_2_‐modified surface. Overall, the mapping results support successful integration of the three constituent components and indicate good compositional homogeneity with limited phase segregation. Although EDS mapping alone cannot definitively resolve the proposed layered architecture or bonding configuration, the observed elemental overlap provides strong complementary evidence for the formation of the intended GO@SiO_2_@[(CH_2_)_3_Im][Cl] nanocomposite.

To synthesize derivatives of 1,2,4,5‐tetraphenyl‐1*H*‐imidazole, we began by optimizing the reaction conditions. As a model system, a one‐pot, four‐component reaction was selected, combining benzil (**1**), benzaldehyde (**2a**), aniline (**3b**), and ammonium acetate (**4**) under microwave irradiation, demonstrating an innovative approach to producing compound (**5a**). This method facilitated a systematic evaluation of several key parameters, as outlined in Table 1. Under the standard 1:1:1:1 stoichiometry of benzaldehyde, aniline, benzil, and ammonium acetate, the imidazolium‐functionalized graphene oxide supported on silica, GO@SiO_2_@[(CH_2_)_3_Im][Cl], proved to be the most effective catalyst. The highest isolated yield (95%) was obtained using 0.0050 g of GO@SiO_2_@[(CH_2_)_3_Im][Cl] with microwave irradiation at 100 W for 10 min at an ambient set temperature of 25 °C. This condition (0.0050 g, 100 W, 10 min), therefore, represents the optimized reaction protocol in the present series of experiments.

**TABLE 1:** Optimization of amount catalyst GO@SiO_2_@[(CH_2_)_3_Im][Cl] and conditions for the synthesis of 1,2,4,5‐tetraphenyl‐1*H*‐imidazole under microwave conditions.

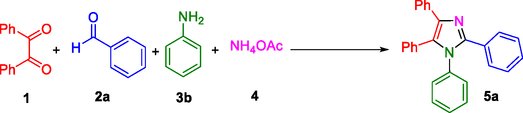					
Entry	Catalyst, g	**Temp., °** **C**	**Microwave** **p** **ower, W**	Time, min	**Yield, %** a
1	No catalyst	25	50	100	No
2	No catalyst	50	75	100	15
3	No catalyst	75	100	100	24
4	GO@SiO_2_@[(CH_2_)_3_Im][Cl] (0.0025)	25	50	10	34
5	GO@SiO_2_@[(CH_2_)_3_Im][Cl] (0.0025)	25	75	10	46
6	GO@SiO_2_@[(CH_2_)_3_Im][Cl] (0.0025)	25	100	10	53
7	GO@SiO_2_@[(CH_2_)_3_Im][Cl] (0.0050)	25	50	10	61
8	GO@SiO_2_@[(CH_2_)_3_Im][Cl] (0.0050)	25	75	10	74
**9**	**GO@SiO**_**2**_ **@[(CH**_**2**_ **)**_**3**_ **Im][Cl] (0.0050)**	**25**	**100**	**10**	**95**
10	GO@SiO_2_@[(CH_2_)_3_Im][Cl] (0.0050)	50	100	10	94
11	GO@SiO_2_@[(CH_2_)_3_Im][Cl] (0.0050)	25	125	10	94
12	GO@SiO_2_@[(CH_2_)_3_Im][Cl] (0.0075)	25	100	10	94
13	GO@SiO_2_@[(CH_2_)_3_Im][Cl] (0.0050)	25	100	15	93
14	GO (0.0050)	25	100	10	39
15	GO@SiO_2_‐Cl (0.0050)	25	100	10	58

*Note:* Conditions: The amount ratios of benzaldehyde, aniline, benzil, and ammonium acetate. [equal to 1:1:1:1], catalyst, solvent‐free.

a Yields referred to isolated products.

A systematic comparison of catalyst loadings revealed a clear loading dependence up to 0.0050 g: at constant microwave power (100 W) and time (10 min), the yield increased from 53% with 0.0025 g to 61% and 74% as the loading was increased to 0.0050 g at lower powers, reaching the maximum 95% at 0.0050 g and 100 W. Further increasing the loading to 0.0075 g did not give a significant improvement (94%), indicating that 0.0050 g is sufficient to achieve near‐quantitative conversion and that higher loadings show diminishing returns.

Microwave power and irradiation time were also important variables. In the absence of a catalyst, extended irradiation (100 min) and elevated temperature gave only low conversion (up to 24%), demonstrating that thermal microwave input alone is insufficient. With GO@SiO_2_@[(CH_2_)_3_Im][Cl], increasing the microwave power from 50 to 100 W at 10 min progressively improved yields (34%–95% for 0.0025–0.0050 g loadings), whereas further increasing the power to 125 W gave no appreciable benefit (94%) and slightly longer irradiation (15 min) produced a marginally lower yield (93%). These observations indicate an optimum balance between microwave power and time at which the catalytic cycle operates most efficiently; excessive power or prolonged exposure does not enhance the outcome and may slightly erode selectivity or promote side processes.

Control experiments using non‐functionalized GO and chloride‐functionalized GO@SiO_2_–Cl, conducted under otherwise identical conditions, underscored the importance of the imidazolium functionality. GO gave only 39% yield while GO@SiO_2_–Cl afforded 58%, both substantially lower than the 95% achieved with GO@SiO_2_@[(CH_2_)_3_Im][Cl]. This enhancement can be rationalized by the combined effects of the silica support and the tethered imidazolium moiety: the supported ionic‐liquid‐like sites likely increase local substrate concentrations, stabilize charged intermediates, and provide acid–base or hydrogen‐bonding interactions that accelerate the multicomponent condensation steps relative to the unmodified materials.

In summary, the optimal, practical protocol identified is solvent‐free microwave irradiation of equimolar benzaldehyde, aniline, benzil, and ammonium acetate in the presence of 0.0050 g GO@SiO_2_@[(CH_2_)_3_Im][Cl] at 100 W for 10 min (ambient set temperature 25 °C), which affords the target imidazole in 95% isolated yield. The data show that both the imidazolium functionalization and the applied microwave conditions are critical to achieving high efficiency and that increasing catalyst loading or microwave power beyond the identified optimum offers no meaningful advantage.

The influence of solvent on the microwave‐assisted multicomponent reaction of benzaldehyde (**1**), aniline (**2a**), and ammonium acetate (**3b**) was systematically investigated under standardized conditions (100 W, 25 °C, 10 min). The results, summarized in the accompanying Table 2, reveal a pronounced solvent dependence, emphasizing the roles of medium polarity and protic character. These solvent properties appear to modulate key steps such as solubilization and proton transfer, which are critical for the reaction's success.

**TABLE 2:** Investigate the influence of solvents in GO@SiO_2_@[(CH_2_)_3_Im][Cl] for the synthesis of 1,2,4,5‐tetraphenyl‐1*H*‐imidazole under microwave conditions.

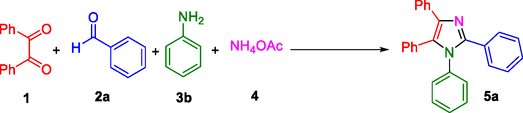						
Entry	Catalyst, g	**Temp.,** °**C**	Solvent	**Microwave** **p** **ower, W**	Time, min	**Yield, %** a
1	**GO@SiO**_**2**_ **@[(CH**_**2**_ **)**_**3**_ **Im][Cl] (0.0050)**	**25**	**Solvent‐free**	**100**	**10**	**95**
2	GO@SiO_2_@[(CH_2_)_3_Im][Cl] (0.0050)	25	H_2_O	100	10	75
3	GO@SiO_2_@[(CH_2_)_3_Im][Cl] (0.0050)	25	EtOH	100	10	66
4	GO@SiO_2_@[(CH_2_)_3_Im][Cl] (0.0050)	25	EtOH:H_2_O (1:1)	100	10	69
5	GO@SiO_2_@[(CH_2_)_3_Im][Cl] (0.0050)	25	CH_2_Cl_2_	100	10	No
6	GO@SiO_2_@[(CH_2_)_3_Im][Cl] (0.0050)	25	CHCl_3_	100	10	No
7	GO@SiO_2_@[(CH_2_)_3_Im][Cl] (0.0050)	25	CH_3_CN	50	10	No

*Note:* Conditions: The amount ratios of benzaldehyde, aniline, benzil, ammonium acetate. [equal to 1:1:1:1], catalyst, solvent (5 mL).

a Yields referred to isolated products.

The most striking outcome was the exceptional efficiency of the solvent‐free condition, which afforded the target imidazole in 95% yield. This high yield under neat conditions suggests that the reaction components interact effectively in the melt phase under microwave irradiation, likely benefiting from enhanced molecular collisions and the absence of solvent dilution effects. The significantly lower yield observed in water (75%) indicates that, while this protic green solvent can support the reaction, it may not optimally solubilize all organic intermediates or facilitate key condensation steps as effectively as the neat medium. A further reduction in yield was observed for ethanol (66%) and a 1:1 ethanol‐water mixture (69%). This trend implies that the purely protic environment of ethanol, potentially combined with its lower boiling point under the experimental conditions, is less conducive to reaction progress than water or the solvent‐free system.

Conversely, the reaction failed in aprotic organic solvents. No product formation was detected in dichloromethane, chloroform, or acetonitrile. This complete inhibition underscores the need for a protic environment, presumably to facilitate proton‐transfer steps integral to the imidazole ring‐forming mechanism. The lack of reactivity in these common organic solvents, despite their ability to dissolve the reactants, strongly suggests that the reaction pathway is not driven solely by conventional heating effects but relies on specific solvent‐solute interactions that are absent in aprotic media.

The solvent screening data demonstrate that the synthesis of **5a** is highly sensitive to the reaction medium. The superior performance under solvent‐free conditions aligns with green chemistry principles by eliminating solvent waste while maximizing efficiency. The failure in aprotic solvents underscores the mechanistic requirement for protic assistance. These findings provide clear practical guidance for optimizing this microwave‐assisted imidazole synthesis, favoring solvent‐free protocols or water as a benign reaction medium.

The influence of substituent electronic and steric properties on the reaction pathway was explored through a systematic experimental approach, which should resonate with researchers valuing robust methods. Using substituted benzaldehyde, substituted aniline, benzil, and ammonium acetate under microwave irradiation at RT, each reaction was catalyzed by 5 mg of a GO@SiO_2_@[(CH_2_)_3_Im][Cl] nanocomposite. As summarized in Table 3, the data indicate a clear trend that aromatic aldehydes bearing electron‐withdrawing groups consistently afforded higher yields and shorter reaction times than their electron‐donating counterparts.

**TABLE 3:** GO@SiO_2_@[(CH_2_)_3_Im][Cl] nanocomposite catalyzed synthesis of 1,2,4,5‐tetraphenyl‐1H‐imidazoles under solvent‐free conditions.

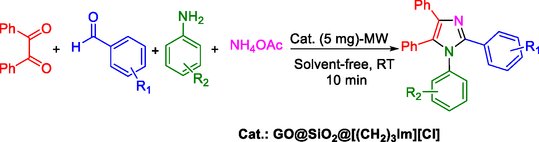			
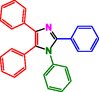	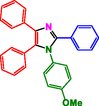	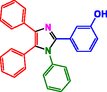	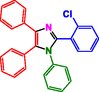
5a, Yields^a^ 95%, M.P. M.P. 219–221 °C [44]	5b, Yields^a^ 96%, M.P. M.P. 174–176 °C [11]	5c, Yields^a^ 93%, M.P. M.P. 186–188 °C [14]	5d, Yields^a^ 92%, M.P. 170–172 °C [6]
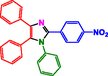	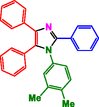	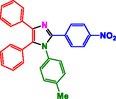	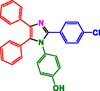
5e, Yields^a^ 97%, M.P. 194–196 °C [66]	5f, Yields^a^ 96%, M.P. 165–167 °C [67]	5g, Yields^a^ 98%, M.P. 219–221 °C [2]	5h, Yields^a^ 97%, M.P. 218–220 °C [16]
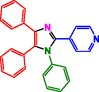	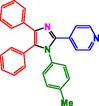	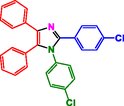	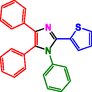
5i, Yields^a^ 91%, M.P. 220–222 °C [33]	5j, Yields^a^ 94%, M.P. 273–275 °C [69]	5k, Yields^a^ 92%, M.P. 185–187 °C [68]	5l, Yields^a^ 90%, M.P. 243–245 °C [33]
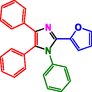	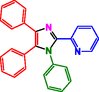	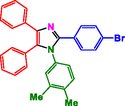	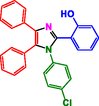
5m, Yields^a^ 91%, M.P. 202–204 °C [6]	5n, Yields^a^ 90%, M.P. 209–211 °C [11]	5o, Yields^a^ 91%, M.P. 143–145 °C [14]	5p, Yields^a^ 92%, M.P. 111–113 °C [44]
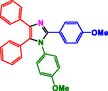	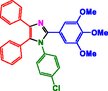		
5q, Yields^a^ 91%, M.P. 203–205 °C [33]	5r, Yields^a^ 90%, M.P. 124–126 °C [68]		

*Note:* Reaction conditions: The amount ratios of substituted benzaldehyde (1 mmol), substituted aniline (1 mmol), benzil (1 mmol), ammonium acetate (1 mmol), and in the presence of GO@SiO_2_@[(CH_2_)_3_Im][Cl] nanocomposite (5 mg) at RT.

a Isolated yield.

**SCHEME 2:**
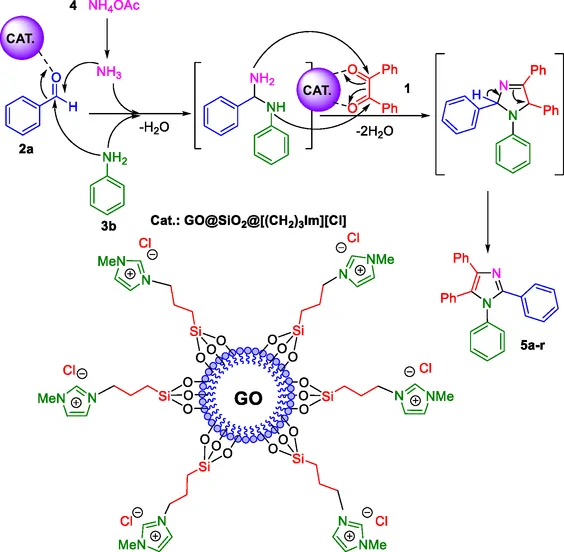
Mechanistic pathway for preparation of 1,2,4,5‐tetraphenyl‐1*H*‐imidazoles.

The sequence shown in Scheme 2 is the familiar Debus–Radziszewski type assembly of a 1,2,4,5‐tetraaryl‐1*H*‐imidazole from a 1,2‐diketone (benzil), an aldehyde (benzaldehyde), and an ammonia source (NH_4_OAc). The mechanism proceeds through a series of nucleophilic additions, condensations, and a final ring closure, followed by dehydration and aromatization; the figure emphasizes the catalytic role of the GO@SiO_2_@[(CH_2_)_3_Im][Cl] material at the key bond‐forming steps.

Mechanistically, the reaction begins with the generation of ammonia from ammonium acetate; that ammonia (or an ammonium/iminium species formed in the presence of the ionic liquid) then attacks the carbonyl electrophiles, promoting imine formation. One plausible and commonly invoked pathway is that ammonia condenses with the aldehyde to form a Schiff base (an imine), while the catalyst simultaneously activates the benzil carbonyls. Activation of a benzil carbonyl by the catalyst increases its electrophilicity and allows nucleophilic attack by the imine nitrogen (or by an ammonia‐derived nitrogen species). This gives a C—N bond and, after a second condensation at the other carbonyl, leads to a bis‐iminium/diamine‐type intermediate. Intramolecular nucleophilic attack and stepwise loss of two molecules of water convert that open‐chain intermediate into a cyclic, partially reduced imidazoline‐type species. Finally, loss of two hydrogen equivalents (dehydrogenation/aromatization) furnishes the aromatic 1,2,4,5‐tetraphenyl‐1*H*‐imidazole product and closes the catalytic cycle.

The GO@SiO_2_@[(CH_2_)_3_Im][Cl] catalyst is multifaceted, responsible for both rate acceleration and the mild, heterogeneous nature of the procedure. The immobilized imidazolium chloride units provide a polar, ion‐pairing environment that facilitates the formation and stabilization of charged intermediates (iminium and ammonium species) and lowers the effective activation barrier for imine and C—N bond formation. The imidazolium cation (and its counter‐anion) can engage carbonyl oxygens by electrostatic and hydrogen‐bonding interactions, thereby increasing carbonyl electrophilicity and directing nucleophilic attack on the diketone. Graphene oxide (GO) brings a high surface area and an abundance of oxygenated functional groups that provide additional hydrogen‐bonding interactions with substrates and intermediates; silica serves as a robust support that disperses the ionic sites and makes the composite mechanically and thermally stable. Taken together, these components create a microenvironment on the catalyst surface that organizes the reacting partners, promotes proton transfers and dehydration steps, stabilizes charged transition states, and facilitates the required redox steps (or at least makes the dehydration/aromatization more favorable under the applied conditions).

Operationally, this manifests as easier imine formation (faster water removal and stabilization of the iminium intermediates), accelerated nucleophilic attack on the activated benzil, and more efficient cyclization and dehydration. Because the catalytic sites are immobilized on GO/SiO_2_, the active species can be recovered and reused, and the heterogeneous catalyst can also limit side reactions by providing a confined, organized surface for multi‐component condensation. In sum, the catalyst functions both as a Lewis/Brønsted activation medium for carbonyl and imine chemistry and as a structured support that promotes assembly, dehydration, and aromatization to efficiently give tetraphenylimidazole.

**FIGURE 9:**
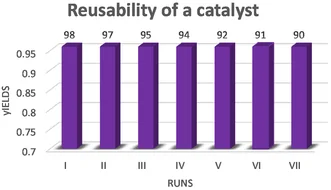
Reusability of GO@SiO_2_@[(CH_2_)_3_Im][Cl] as a catalyst for the synthesis of 1,2,4,5‐tetraphenyl‐1*H*‐imidazoles.

Figure 9 presents the reusability study of GO@SiO_2_@[(CH_2_)_3_Im][Cl] as a heterogeneous catalyst for the synthesis of 1,2,4,5‐tetraphenyl‐1*H*‐imidazoles. Seven consecutive runs (I through VII) were performed under identical reaction conditions, and the isolated yields were recorded for each cycle. The data reveal exceptionally high catalytic performance across all seven uses, with yields of 98%, 97%, 95, 94%, 92%, 91%, and 90%, respectively. The results indicate that the solid catalyst maintains substantial activity upon repeated deployment, with each reuse delivering products at yields >90%.

From a methodological standpoint, the seven‐run sequence demonstrates a modest, monotonic decline in activity. The initial yield is 98% and the final yield after the seventh cycle is 90%, representing an overall decrease of 8 percentage points (approximately an 8% relative loss) over seven cycles. This pattern suggests that the catalyst is robust but gradually deactivates with successive uses. The consistent high performance in early cycles highlights the effectiveness of immobilizing the imidazolium chloride moiety on the GO@SiO_2_ support, while the gradual drop points to slow deactivation mechanisms that warrant further investigation.

Several factors could contribute to the observed deactivation. Leaching or partial detachment of the active imidazolium sites from the support could reduce the number of available catalytic sites over time. Surface fouling by reaction byproducts or partial pore blockage could also hinder substrate access to the active centers. Additionally, minor material loss during the recovery and washing steps between runs cannot be entirely ruled out. However, the magnitude of activity loss is modest, underscoring the practical recyclability of the catalyst for iterative synthesis.

For a comprehensive assessment of stability, complementary analyses would be valuable. Hot filtration tests and leaching studies could quantify the extent of migration of any active species into the reaction medium. Post‐cycle characterization using techniques such as FT‐IR, XPS, and TEM would help ascertain whether functional groups, the ionic–liquid–type moieties, or the morphology of the GO/SiO_2_ support undergo significant changes during reuse. Inductively coupled plasma analysis could detect trace metals or other leachates, providing a clearer picture of the heterogeneity of the catalysis.

When compared with related heterogeneous systems for imidazole synthesis, the GO@SiO_2_@[(CH_2_)_3_Im][Cl] catalyst combines high initial activity with notable recyclability, delivering consistently high yields over multiple cycles and enabling straightforward product isolation due to its solid‐supported nature. The observed slight deactivation, while not negligible, remains within acceptable bounds for practical applications and suggests that modest optimization—such as refined washing procedures, stronger immobilization strategies, or protective surface treatments—could further enhance long‐term stability.

**FIGURE 7:**
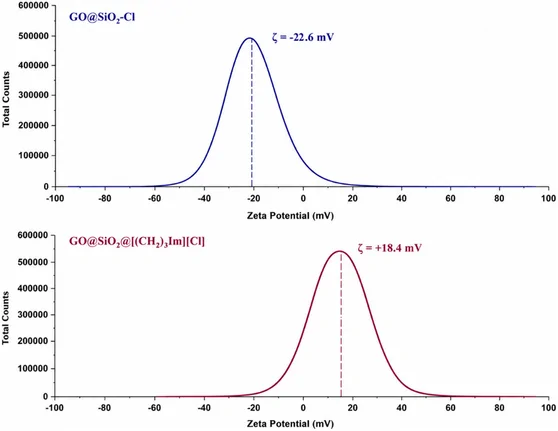
Zeta‐potential analysis of the GO@SiO_2_@[(CH_2_)_3_Im][Cl] nanocomposite.

The data illustrated in Figure 7 demonstrate that GO@SiO_2_@[(CH_2_)_3_Im][Cl] functions as a highly active and recyclable catalyst for the targeted multicomponent synthesis of 1,2,4,5‐tetraphenyl‐1H‐imidazoles. The seven‐cycle performance, with yields remaining in the 90%–98% range, supports its potential utility in sustainable, scalable synthesis, while the observed gradual deactivation highlights opportunities for further improvement through mechanistic and materials‐stability investigations.

**FIGURE 10:**
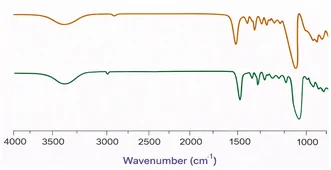
A comparison of the FT‐IR spectra between the fresh (up) and reused (down) catalyst after 7 runs.

Figure 10 presents a direct FT‐IR comparison of the fresh GO@SiO_2_@[(CH_2_)_3_Im][Cl] catalyst (upper/orange trace) and the same material after seven reuse cycles (lower/green trace). The spectra are remarkably similar across the full 4000–1000 cm^−1^ range, indicating that the principal chemical architecture—comprising the silica/support framework, the grafted imidazolium‐like moieties, and the GO/oxide interface—remains intact mainly after repeated use. In both spectra, bands assignable to the Si‐O‐Si network are evident in the 1100–1000 cm^−1^ region, which corroborates the persistence of the silica‐based support. The presence of C–H stretches around 2900 cm^−1^ and aromatic/imidazolium‐associated bands in the mid‐ to lower‐wave number region (approximately 1600–1400 and 1200–1000 cm^−1^) further supports the continued existence of the immobilized ionic–liquid–type functionalities on the GO@SiO_2_ scaffold.

Comparing the two spectra reveals only minor, systematic differences. The reused catalyst shows a slight reduction in the intensity of bands that can be attributed to the imidazolium‐derived moieties, particularly in the regions associated with C=C/C=N vibrations (roughly 1560–1450 cm^−1^) and the C–N/C–O–C–related bands around 1180–1100 cm^−1^. These attenuations are consistent with a small fraction of functional groups becoming less accessible or partially lost during the recycling process, without implying wholesale degradation of the catalyst's core framework. In addition, the reused spectrum exhibits a faint, broad feature near 3400–3500 cm^−1^, commonly assigned to adsorbed moisture or surface hydroxyl groups, and is often enhanced after multiple handling and washing steps. Crucially, no new absorptions are observed in regions such as the carbonyl/oxide‐sensitive zone (e.g., near 1700 cm^−1^), suggesting that no major chemical transformations—such as oxidation or carbonate formation—occurred during reuse.

Taken together, the FT‐IR data indicate that the catalyst retains its essential structural integrity after seven cycles, which aligns well with the observed high catalytic performance and the modest decline in activity reported in the reuse study. The small changes in band intensity and the appearance of only routine surface‐moisture features imply that, if any, deactivation arises mainly from surface‐site accessibility or minor leaching/fouling rather than from catastrophic chemical degradation of the support or immobilized moieties. For a more definitive picture of the deactivation mechanisms, complementary analyses—such as XPS to track surface composition, TGA to quantify any loss of organic content, and post‐reaction TEM or SEM to assess morphological changes—would be valuable. The FT‐IR results corroborate the conclusion that GO@SiO_2_@[(CH_2_)_3_Im][Cl] is a robust, recyclable catalyst, with only modest changes observed after seven reuse cycles, consistent with its demonstrated high‐yield performance in repeated runs.

**FIGURE 11:**
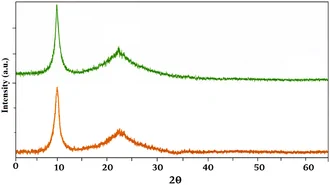
A comparison of the XRD spectra between the fresh (down) and reused (up) catalyst after 7 runs.

Figure 11 provides a direct comparison of the XRD patterns of the fresh GO@SiO_2_@[(CH_2_)_3_Im][Cl] catalyst (orange trace, referred to as the down or fresh sample) and the same material after seven reuse cycles (green trace, referred to as the up or reused sample). The spectra were collected over a 2*θ* range from 0° to about 60°, capturing the characteristic diffraction features of the GO/SiO_2_ support and the immobilized ionic‐liquid‐like moieties embedded in the graphene oxide‐silica framework.

Visually, the two patterns are dominated by relatively broad features rather than sharp, well‐defined Bragg peaks, consistent with a mostly amorphous silica matrix and the planar, disordered nature of GO domains. In both traces, no sharp new reflections emerge upon reuse, and the significant features appear at nearly the same 2*θ* positions, indicating that the catalyst's principal crystalline framework is retained after seven cycles. The down (fresh) spectrum and the up (reused) spectrum thus reflect a preserved structural backbone rather than the formation of new crystalline phases.

Nevertheless, subtle differences in intensity are discernible between the two patterns. The regenerated catalyst exhibits only modest changes in the relative intensities of the principal features: a slight modulation in the low‐angle region (around 9°–12° 2*θ*) and a marginal adjustment of the broader features in the 20°–25° region. These variations are slight and fall within typical experimental variability, suggesting that there is no substantial restructuring of the framework or loss of long‐range order during reuse. Crucially, no new peaks are observed anywhere in the spectrum, which would be indicative of crystalline decomposition products, phase transitions, or the incorporation of metallic or carbonate species due to repeated use and washing.

Taken together, the XRD data in Figure 9 corroborate the conclusion drawn from the other characterization modalities: the GO@SiO_2_@[(CH_2_)_3_Im][Cl] catalyst maintains its essential crystalline framework after seven reuse cycles. The absence of new crystalline phases and the preservation of the overall diffraction pattern imply that the core support—comprising silica and GO domains—and the immobilized ionic–liquid–type functionalities remain intact to a first approximation. This structural robustness aligns with the observed high catalytic performance over multiple runs and with the FT‐IR results, which likewise indicated only modest surface‐level changes rather than wholesale degradation.

From a mechanistic standpoint, the retained XRD pattern supports the interpretation that any deactivation observed during recycling is more likely attributable to surface phenomena—such as modest leaching or fouling of accessible catalytic sites, or slight changes in surface area and porosity—rather than to deterioration of the underlying GO/SiO_2_ scaffold or to irreversible chemical transformation of the immobilized moieties. To more precisely delineate the nature of any subtle changes, complementary analyses would be valuable, including X‐ray photoelectron spectroscopy to monitor surface composition and oxidation states, thermogravimetric analysis to quantify any loss of organic content, and electron microscopy to assess potential nanoscale morphological changes after repeated use. The XRD data support the core conclusion that the catalyst remains structurally robust and chemically stable across seven reuse cycles, consistent with its demonstrated high activity and recyclability.

### 2.3 Hot Filtration Test

**FIGURE 12:**
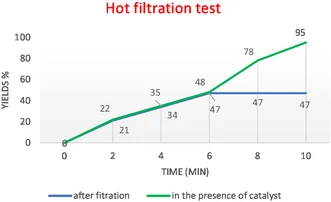
Leaching experiment of GO@SiO_2_@[(CH_2_)_3_Im][Cl] nanocatalyst on the model reaction (product **5a**).

A hot‐filtration (leaching) experiment was performed to probe whether the catalytic activity of the GO@SiO_2_@[(CH_2_)_3_Im][Cl] nanocatalyst in the model transformation to product **5a** derived from a truly heterogeneous surface‐bound species or from catalytically active material leached into solution (Figure 12). Reaction progress was followed by measuring yield (%) at 2‐minute intervals up to 10 min. At *t* = 6 min, the solid catalyst was removed by hot filtration, and the filtrate was allowed to react further; yields were recorded for both the uninterrupted reaction in the presence of the solid catalyst and the filtrate after removal of the solid catalyst.

With the solid catalyst present, the reaction yield increased steadily from 0% at *t* = 0 min to 22% at 2 min, 35% at 4 min, 48% at 6 min, 78% at 8 min, and 95% at 10 min. After removal of the solid catalyst at 6 min, the filtrate yield showed the same early‐time behavior. It reached 47% at the moment of filtration (0%, 21%, 34%, and 47% at *t* = 0, 2, 4, and 6 min, respectively), but thereafter it remained essentially unchanged: the yield stayed at approximately 47% at both 8 and 10 min. Thus, beyond the 6‐minute point at which the solid was removed, the reaction stalled in the absence of the solid catalyst. In contrast, the parallel experiment with the solid present reached near‐quantitative conversion (95%) within 10 min.

The close agreement of the two traces up to the filtration point indicates that the initial rate is unaffected by the impending removal, but the immediate cessation of conversion after catalyst removal demonstrates that no appreciable amount of catalytically active species is present in the filtrate. In other words, the hot‐filtration test provides strong evidence that the activity of this system arises from the heterogeneous GO@SiO_2_@[(CH_2_)_3_Im][Cl] surface rather than from homogeneous leached species. The rapid increase in yield observed only when the solid remains in contact with the reaction medium supports the conclusion that the active sites are retained on the solid support under the applied conditions. Complementary analyses (for example, elemental analysis or ICP of the filtrate) would provide additional confirmation by directly quantifying any trace leaching. However, the present kinetic behavior already indicates good heterogeneity and operational stability of the nanocatalyst under the tested reaction conditions.

Table 4 summarizes a comparative survey of catalytic systems reported for the synthesis of 1,2,4,5‐tetraphenyl‐1*H*‐imidazoles and includes the activity of the GO@SiO_2_@[(CH_2_)_3_Im][Cl] nanocomposite developed in the present work. The entries are as follows. Entry 1: SBA–Pr–SO_3_H under solvent‐free conditions at 140 °C afforded a 78% yield after 240 min. Entry 2: 1‐Butyl‐3‐methylimidazolium bromide ([Bmim]Br) at 140 °C gave a 91% yield in 150 min. Entry 3: BF_3_·SiO_2_ under solvent‐free conditions at 140 °C delivered a 92% yield in 120 min. Entry 4: MgAl_2_O_4_ nanoparticles (0.05 g) used with ultrasound irradiation in ethanol at 60 °C provided a 91% yield in 15 min. Entry 5: LADES@MNP (7.5%) under solvent‐free sonication at RT produced a 73% yield in 150 min. Entry 6: InCl_3_·3H_2_O in methanol at RT required 531 min to give an 83% yield. Entry 7: a DMU: SnCl_2_·HCl system at 90 °C reached a 95% yield in 110 min. Entry 8 (present work): the GO@SiO_2_@[(CH_2_)_3_Im][Cl] nanocomposite under solvent‐free microwave irradiation at RT achieved a 95% yield in 10 min. Entry 8: Microwave irradiation further reduced processing times: Amberlyst A‐15 under solvent‐free microwave conditions afforded 87% in 12 min, albeit at a relatively high catalyst mass (350 mg).

**TABLE 4:** A comparison of the activity of GO@SiO_2_@[(CH_2_)_3_Im][Cl] nanocomposite with various catalysts reported in the literature for the synthesis of 1,2,4,5‐tetraphenyl‐1*H*‐imidazoles.

Entry	Condition	Time, min	Yields, %	Ref.
1	SBA‐Pr‐SO_3_H, Solvent free/140 °C	240	78	[67]
2	[Bmim]Br, 140 °C	150	91	[2]
3	BF_3_.·SiO_2_, Solvent free/140 °C	120	92	[6]
4	MgAl_2_O_4_ (0.05 g) NPs, Ultrasound irradiation, EtOH, 60 °C	15	91	[68]
5	LADES@MNP (7.5%), solvent‐free sonication, RT	150	73	[69]
6	InCl_3_⋅·3H_2_O, RT/MeOH	531	83	[33]
7	DMU: SnCl_2_:HCl, 90 °C	110	95	[70]
8	Amberlyst A‐15 (350 mg), Solvent free, MW	12	87	[74]
**9**	**GO@SiO** **_2_** **@[(CH** **_2_** **)** **_3_** **Im][Cl] (5 mg), Solvent free, MW (100), RT**	**10**	**95**	**Pres** **ent work**

Comparison of these results highlights the combined advantages of the GO@SiO_2_@[(CH_2_)_3_Im][Cl] catalyst, including both reaction rate and isolated yield. The GO‐based nanocomposite affords the highest reported yield (95%) while also producing the product in the shortest time (10 min), representing a marked acceleration relative to most literature systems. The closest competitor in overall speed is the MgAl_2_O_4_ nanoparticle system, which reaches a comparable yield (91%) in 15 min but requires ultrasound activation and operation at 60 °C in ethanol; by contrast, the GO@SiO_2_@[(CH_2_)_3_Im][Cl] protocol proceeds solvent‐free at RT under microwave irradiation. Several previously reported catalysts obtain high yields but require substantially longer reaction times and/or elevated temperatures: BF_3_·SiO_2_ and [Bmim]Br required 120–150 min at 140 °C to achieve 91%–92% yields. The DMU: SnCl_2_·HCl system achieves the exact final yield (95%) but only after 110 min at 90 °C. Other methods balance milder temperatures with extended reaction times, as illustrated by InCl_3_·3H_2_O in methanol at RT, which yields 83% after 531 min. The LADES@MNP approach operates at RT under sonication but gives a lower yield (73%) and a lengthy reaction time (150 min).

The superior performance of the GO@SiO_2_@[(CH_2_)_3_Im][Cl] material likely reflects synergistic contributions from its structural and chemical features. The graphene oxide support offers a high surface area. It facilitates dispersion of active sites, the silica spacer can prevent aggregation and improve accessibility, and the immobilized imidazolium moiety may provide both Lewis/Brønsted acidity and ionic microenvironments that promote the multicomponent condensation pathway. In addition, microwave irradiation is known to accelerate reactions through rapid volumetric heating and selective activation of polar reaction components; in the present solvent‐free system, these effects will enhance intermolecular encounters and may create localized “hot spots” at the catalyst interface, thereby dramatically reducing reaction times without compromising yields. Compared with ultrasound‐assisted or conventional thermal activations, the microwave protocol combines mild bulk conditions (RT) with very high apparent reaction rates.

While these comparative data underscore clear practical advantages—short reaction times, high isolated yields, solvent‐free operation, and low bulk temperature—the broader application of the GO@SiO_2_@[(CH_2_)_3_Im][Cl] system should be evaluated with respect to catalyst recyclability, long‐term stability, substrate scope, and scalability under continuous or larger‐scale microwave conditions. Microwaves can be challenging to scale uniformly, and potential limitations arising from microwave penetration depth or catalyst heating heterogeneity merit further study. Nevertheless, within the set of catalysts surveyed here, the GO@SiO_2_@[(CH_2_)_3_Im][Cl] nanocomposite provides the most favorable combination of efficiency and green‐chemistry attributes reported to date.

## 3 Experimental

All chemicals used in this study were sourced from Merck, Fluka, and Aldrich and used as received without further purification to ensure reproducibility.

Reaction progress was routinely monitored by thin‐layer chromatography (TLC) on precoated silica gel plates (PolyGram SIL G/UV 254). Product identities and purities were confirmed by nuclear magnetic resonance (NMR) spectroscopy to validate the synthesis process.

### 3.1 Preparation of GO@SiO_2_@[(CH_2_)_3_Im][Cl] Nanocomposite

The synthesis of the GO@SiO_2_@[(CH_2_)_3_Im][Cl] nanocomposite was achieved via a three‐step sequential procedure, beginning with the functionalization of graphene oxide (GO). In the first step, GO (5 mg) was converted to GO‐Cl by refluxing in a mixture of thionyl chloride (20 mL) and dimethylformamide (60 mL) at 100 °C under a nitrogen atmosphere for 24 h. Excess SOCl_2_ was removed by distillation, and the resulting solid was washed with dimethylacetamide (DMAc), filtered, and dried under vacuum at 80 °C to ensure consistent functionalization.

This GO‐Cl intermediate (0.5 g) was then used to synthesize the GO@SiO_2_@[(CH_2_)_3_Im][Cl]. The GO‐Cl was combined with 1‐methyl‐1*H*‐imidazole (1 mmol) in dichloromethane (40 mL). The suspension was sonicated for 30 min to ensure homogeneous dispersion and then refluxed with vigorous stirring for 12 h. The product was separated, washed with dichloromethane, and vacuum‐dried to yield the GO@SiO_2_@[(CH_2_)_3_Im][Cl].

### 3.2 General Protocol for the Preparation of 1,2,4,5‐Tetrasubstituted Imidazoles Using GO@SiO_2_@[(CH_2_)_3_Im][Cl] Nanocomposite

In the experimental procedure, a mixture consisting substituted benzaldehyde (1.0 mmol), substituted aniline (1.0 mmol), benzil (1.0 mmol) and ammonium acetate (1.0 mmol) was combined with GO@SiO_2_@[(CH_2_)_3_Im][Cl] nanocomposite (5 mg) reaction vial. The vial was hermetically sealed and pressurized for 20 s before subjecting it to microwave irradiation at 25 °C with a power output of 100 W in 10 min. The reaction progress was monitored via thin‐layer chromatography (TLC) using an n‐hexane–ethyl acetate (3:1) solvent system to ensure complete consumption of the starting materials.

Once the reaction was completed, the mixture was allowed to cool at RT for 10 min. The solid product was then filtered out, and the residue was dissolved in 3 mL of hot ethanol. The catalyst was recovered for reuse through centrifugation and subsequently washed with ethanol and dried in an oven. Upon completion, the reaction mixture was cooled to RT. Upon completion of the reaction, the ethyl acetate (10 mL) and water (10 mL) were added to the reaction mixture. The resulting mixture was then separated into a water and an organic phase. The ethyl acetate was then removed by rotary evaporation to isolate crude 1,2,4,5‐tetrasubstituted imidazole derivatives. The crude products were further recrystallized in ethanol.

### 3.3 Novelty

The principal novelty of this work lies in the design and application of a previously unreported heterogeneous nanocomposite, GO@SiO_2_@[(CH_2_)_3_Im][Cl], which uniquely integrates the advantageous properties of graphene oxide, silica, and an ionic liquid moiety into a single catalytic architecture for the synthesis of 1,2,4,5‐tetrasubstituted imidazoles. While individual components or simpler hybrids have been employed in similar transformations, the strategic covalent anchoring of the chloro‐functionalized ionic liquid, 1‐methyl‐3‐propylimidazolium chloride, onto a silica‐coated graphene oxide surface represents an unprecedented synergistic assembly. This specific structural configuration not only enhances the dispersion and stability of the catalytic sites but also creates a microenvironment that facilitates the four‐component condensation under exceptionally mild conditions. The ability of this novel nanocomposite to promote the reaction at room temperature under solvent‐free conditions with microwave assistance, achieving near‐quantitative yields (90%–98%) in just 10 min across a broad substrate scope (18 examples), is a significant departure from existing methodologies. These often require elevated temperatures, extended reaction times, or the use of volatile organic solvents, even when employing other nanocatalysts. Therefore, the core novelty is twofold: the synthesis of a new, sophisticated nanocomposite material with a tailored structure, and its demonstration as a highly efficient, sustainable, and rapid platform for this important heterocyclic synthesis, thereby establishing a new benchmark for the protocol.

## 4 Conclusion

In conclusion, we have developed a highly efficient and environmentally benign protocol for the one‐pot, four‐component synthesis of 1,2,4,5‐tetrasubstituted imidazoles. The methodology leverages the synergistic properties of a novel GO@SiO_2_@[(CH_2_)_3_Im][Cl] nanocomposite, which serves as a robust, reusable heterogeneous catalyst. The reaction, conducted under solvent‐free conditions at room temperature using microwave irradiation, reached completion in just 10 min, affording the desired products in excellent yields of 90%–98%. The versatility and general applicability of this approach were demonstrated across 18 diverse examples, accommodating various substituted anilines and benzaldehydes. The combination of ambient temperature, solvent‐free conditions, and rapid microwave‐assisted synthesis underscores the significant advantages of this method, including operational simplicity, energy efficiency, and alignment with green chemistry principles. The exceptional catalytic performance is attributed to the unique structural and chemical features of the nanocomposite, which effectively facilitate the reaction through synergistic interactions among its components. This work not only provides a robust and sustainable alternative for the synthesis of this important class of heterocycles but also opens new avenues for the design and application of graphene‐based nanocomposites in multicomponent reactions.

## 5 Future Outlook

Building upon the promising results obtained with the GO@SiO_2_@[(CH_2_)_3_Im][Cl] nanocomposite, several avenues for future investigation emerge from this work. A comprehensive mechanistic study employing in situ spectroscopic techniques and kinetic analysis would provide valuable insights into the precise role of each component of the catalytic system, particularly the synergistic interplay between the graphene oxide support, the silica spacer, and the ionic liquid functionality. Such understanding could inform the rational design of even more efficient second‐generation catalysts. Furthermore, a detailed investigation into the long‐term reusability and stability of the nanocomposite under continuous‐flow conditions would be a logical progression, assessing its potential for industrial‐scale applications. Given the mildness and efficiency of the developed protocol, expanding the substrate scope to include structurally diverse and sterically demanding amines, aldehydes, and even alternative 1,2‐dicarbonyl compounds could further demonstrate the method's generality and utility in constructing libraries of biologically relevant imidazole derivatives. Finally, exploring the application of this nanocomposite in other multicomponent reactions for the synthesis of diverse heterocyclic scaffolds would underscore its broader utility as a versatile catalytic platform in sustainable organic synthesis.

## Conflicts of Interest

The authors declare no conflicts of interest.

## Data Availability

Data sharing not applicable to this article as no datasets were generated or analysed during the current study.
